# Differences in archaeal diversity and potential ecological functions between saline and hypersaline lakes on Qinghai-Tibet Plateau were driven by multiple environmental and non-environmental factors beyond the salinity

**DOI:** 10.1186/s12866-024-03307-3

**Published:** 2024-05-04

**Authors:** Yaqiong Wang, Wenxin Li, Guoyuan Bao, Mohan Bai, Huike Ye

**Affiliations:** 1 School of Ecology, Environment and Resources, Qinghai Minzu University, Bayi Road, Xining, 810007 Qinghai China; 2 Agro-Environmental Protection Institute, Ministry of Agriculture and Rural Affairs / Key Laboratory of Original Agro-Environmental Pollution Prevention and Control, MARA / Tianjin Key Laboratory of Agro-Environment and Agro-Product Safety, Tianjin, 300191 China; 3 Qinghai Provincial Key Laboratory of High-Value Utilization of Characteristic Economic Plants, Xining, 810007 China; 4 Qinghai Provincial Biotechnology and Analytical Test Key Laboratory, Xining, 810007 China

**Keywords:** Archaea, Diversity, Ecological functions, Plateau saline lakes, Network analysis

## Abstract

**Background:**

Saline lakes are home to various archaea that play special and crucial roles in the global biogeochemical cycle. The Qinghai-Tibet Plateau hosts a large number of lakes with diverse salinity ranging from 0.1 to over 400 g/L, harboring complex and diverse archaea. To the best of our knowledge, the formation mechanisms and potential ecological roles of archaea in Qinghai-Tibetan Plateau saline lakes remain largely unknown.

**Results:**

Using High-throughput Illumina sequencing, we uncovered the vastly distinct archaea communities between two typical saline lakes with significant salinity differences on the Qinghai Tibet Plateau (Qinghai saline lake and Chaka hypersaline lake) and suggested archaea played different important roles in methanogenesis-related and nitrate reduction-related functions of these two lakes, respectively. Rather than the individual effect of salinity, the composite effect of salinity with diverse environmental parameters (e.g., temperature, chlorophyll a, total nitrogen, and total phosphorus) dominated the explanation of the variations in archaeal community structure in different habitats. Based on the network analysis, we further found the correlations between dominant archaeal OTUs were tight but significantly different between the two habitats, implying that archaeal interactions may also largely determine the shape of archaeal communities.

**Conclusion:**

The present study improved our understanding of the structure and function of archaea in different saline lakes on the Qinghai-Tibet Plateau and provided a new perspective on the mechanisms underlying shaping their communities.

**Supplementary Information:**

The online version contains supplementary material available at 10.1186/s12866-024-03307-3.

## Background

Saline lakes are found all over the world and play important roles in global biogeochemical cycling [1, 2]. Although all three domains of life (Archaea, Bacteria, and Eukarya) could be found in saline lakes, archaea were thought to represent the unneglectable important component of the biological community in saline lakes, particularly in some hypersaline lakes (salinity > 50.0 g/L) [3–6]. For example, in the high-salinity sediments of hypersaline lake Chaka, the abundance of archaea was found two times higher than bacteria [5]. In the Great Salt Lake (salinity > 240.0 g/L) waters, more than 500 archaea sequences were detected using the clone-library method, which were attributed to 4 known genera (*Haloplanus*, *Halorubrum*, *Natronococcus*, *Haloquadratum*) and 5 unknown groups [6]. Comparatively, only a few bacteria sequences were detected in the Great Salt Lake at the same time, and almost all of them belonged to the bacterial genus *Salinibacter*. Furthermore, some previous studies discovered different taxa of archaea presented different metabolic characteristics, implying that they played varied ecological functions in the environment [7–9]. Thus, it is important to generate information on the archaea communities and ecological functions present in different saline lakes, as well as how they are influenced by their surroundings.

Salinity has frequently been assumed to be the primary important environmental factor influencing the composition of microorganisms in different saline lakes [5, 6]. For the archaea, a few studies have also revealed that salinity was a significant factor in the formation of their community in saline lakes, such as Gahai, Xiaochaidan, and Charhan lake [10, 11]. However, more and more recent evidences suggested the explanation of salinity on microorganisms’ community variations in different saline lakes was still very limited. For example, in saline lakes with different salinity on the Qinghai Tibet Plateau, it has been reported that salinity can only explain 14–33% of the bacterial community variations [12]. On one hand, these unexplained variations can be attributed to the unmeasured environmental factors [13, 14]. On the other hand, microbial interactions have recently been proposed to be an alternative explanation [15, 16]. However, up to our knowledge, how the environmental factors and microbial interactions influence the archaeal communities in different saline lakes still remains largely unexplored.

The Qinghai-Tibet Plateau hosts a large number of lakes with diverse salinity ranging from 0.1 to over 400 g/L, making it a natural laboratory and resource treasure house for studying archaea. However, although a few studies were used to study archaea diversity in different Qinghai-Tibet Plateau saline lakes [5, 11], the formation mechanisms and potential ecological roles of archaea in these lakes remain largely unknown. In this study, we performed 16 S rRNA gene phylogenetic analyses in two typical salinity lakes on the Qinghai-Tibet Plateau: Qinghai saline lake and Chaka hypersaline lake, which were nearly in graphic and altitude but significantly different in salinity. The ultimate goal of this study was to improve our understanding of the structure and potential ecological function of archaea in different plateau saline lakes, as well as to identify the dominant driving factors of the archaea communities.

## Methods

### Sample collection

Water samples were collected from two lakes in July 2022. Qinghai saline lake samples were marked as QW1, QW2, QW3, QW4, QW5, and QW6, and Chaka hyper-saline lake samples were marked as CW1, CW2, CW3, CW4, and CW5 (Table S1). Two liters of water were collected from each sampling site and stored in sterile containers to prevent contamination. One liter of water in individual stations was filtered through 0.22 μm polycarbonate Ispore membrane filters (Millipore, USA) and stored at -80 °C for sequencing analysis. The remaining water was analyzed for environmental variables within 24 h of collection.

### Analysis of environmental variables

Dissolved oxygen (DO), pH, and salinity data were monitored on-site using YSI Pro Plus sensors (YSI, Yellow Springs, OH, USA). Ammonium (NH_4_^+^), nitrate (NO_3_^−^), and phosphate (PO_4_^3−^) levels were measured using a QuAAtro39 Continuous Segment-ed Flow Analyzer (SEAL Analytical, Inc., Mequon, WI, USA). Chlorophyll a (Chl_a), total nitrogen (TN), and total phosphate (TP) were measured following the methods described by He [17]. The results are summarized in Table S1.

### DNA extraction and amplicon sequencing

Total genomic DNA of environmental samples was extracted from 0.22-µm filters stored at -80 °C using an E.N.Z.A.™ Water DNA Kit (Omega Biotek, Inc., Norcross, GA, USA). The isolated DNA pellet was resuspended in 100 µL of Milli-Q water (Merck Millipore, Burlington, MA, USA), quantified using a Nanodrop Spectrophotometer (Thermo Fisher Scientific, Waltham, MA, USA), and stored at -20 °C until further use. To increase the recovery rate and specificity of archaeal sequences during high-throughput sequencing, two rounds of nested PCR were used in this study. For the first round, the archaeal-specific primers GU1ST-340 F (5′-CCCTAYGGGGYGCASCAG-3′) and GU1ST-1000R (5′-GGCCATGCACYWCYTCTC-3′) [18] were used within the following program: a denaturation step at 95 °C for 3 min, followed by five cycles consisting of 30 s at 94 °C, 20 s at 45 °C, 30 s at 65 °C; 20 cycles consisting of 20 s at 94 °C, 20 s at 55 °C, 30 s at 72 °C, a final extension at 72 °C for 5 min. 20–30 ng of the PCR product from the first round was used as template DNA for the second PCR, which was conducted using the same PCR conditions and the general V3–V4 primer sets 349 F (5′-GYGCASCAGKCGMGAAW-3′) and 806R (5′-GG′ACTACVSGGGTATCTAAT-3′) [19]. PCR products were examined by 2% (w/v) agarose gel electrophoresis in (Tris, Boric acid, TBE EDTA) buffer solution. Subsequently, the amplicons were loaded onto an Illumina HiSeq platform (Illumina Inc., San Diego, CA, USA) according to the manufacturer’s guidelines.

### Bioinformatics analysis

The fastq file that resulted from the interpreted flowgrams was deposited in the NCBI Sequence Read Archive (BioProject number PRJNA983497). After sequencing, Cutadapt (v.1.18) [20] was used to remove the primer adapter and the read pairs were sutured (merged) into sequences using PEAR (v.0.9.8) [21], based on the overlapping relationship between PE read segments. FASTQ files were processed to generate separate FASTA and QUAL files, which were then analyzed using standard methods. PRINSEQ (v.0.20.4) [22] was used to excise bases with a mass value below 20 at the tail of the reads, and a window of 10 bp was set if the mean mass value within the window was below 20. The back-end bases were cut off from the window, sequences containing N and short sequences were filtered after quality control, and sequences with low complexity were filtered out. The FASTA files were de-replicated, abundance sorted, and singleton sequences were removed. The OTUs (using a 97% similarity cut-off) of archaea were clustered de novo using USEARCH (v.11.0.667) [23]. Mothur (1.43.0) [24] was used to determine the alpha diversity index. The Redundancy, Venn, Mantel-test, and network analysis of different samples were performed using R (v.4.3.0) software. Archaeal metabolism functional prediction was conducted using PICRUSt (v.1.1.4) [25, 26]. The ecological functional prediction of the archaeal communities was conducted using FAPROTAX (v1.2.1) [27].

## Results

### Differences in archaeal diversity, taxonomic compositions, and potential ecological functions

The overall number of archaeal sequences in Qinghai saline lake and Chaka hypersaline lake was 233,097 and 179,359, which were clustered into 791 and 428 operational taxonomic units (OTUs, at a 97% cut-off), respectively. As Table 1 shown, the richness (Chao index) of archaea in Qinghai saline lake (in an average of 532) was significantly higher (Wilcox.test, *p* = 0.004 < 0.05) than that in Chaka hypersaline lake (in an average of 352), suggesting more archaeal species were adaptable to the saline lake, but sensitive to the hypersaline environments. However, the alpha diversity (both Shannon and Simpson Index) in Chaka hypersaline lake showed no significant difference (Wilcox.test, *p =* 0.823 and 0.123 > 0.05) with that of Qinghai saline lake, demonstrating a stable and highly adapted archaea community existed in the hypersaline lake. Using Principal coordinate analysis (PCoA) with Bray-Curtis distance, we compared the beta diversity of archaeal communities in different lakes. Based on our results, the first two principal components explained 53.9% and 11.5% of the total variation, respectively. Furthermore, the PERMANOVA (*p* = 0.002 < 0.005) results and PCoA ordination plots indicated that there were significant differences in the composition of archaea between the two lakes (Fig. S1).

**Table 1:** Statistical analysis of archaea alpha diversity in different habitats

Habitat	Sample	Sequences	OTUs Number	Shannon	Chao	Simpson
Chaka hypersaline lake	CW1	42,029	261	3.523	369.966	0.054
	CW2	37,242	246	3.471	363.300	0.066
	CW3	38,485	207	3.063	308.077	0.076
	CW4	20,847	253	3.069	342.375	0.085
	CW5	40,756	233	3.404	375.375	0.097
Mean ± sd		35,872 ± 7697	240 ± 19	3.306 ± 0.200	351.818 ± 24.571	0.076 ± 0.015
Qinghai saline Lake	QW1	22,283	417	3.846	558.915	0.050
	QW2	38,077	494	4.002	689.735	0.038
	QW3	43,233	409	2.763	499.833	0.169
	QW4	45,293	355	3.775	472.903	0.051
	QW5	40,067	426	3.875	568.600	0.063
	QW6	44,144	271	3.754	400.636	0.036
Mean ± sd		38,850 ± 7803	395 ± 69	3.669 ± 0.413	531.770 ± 90.118	0.068 ± 0.046

**Fig. 1:**
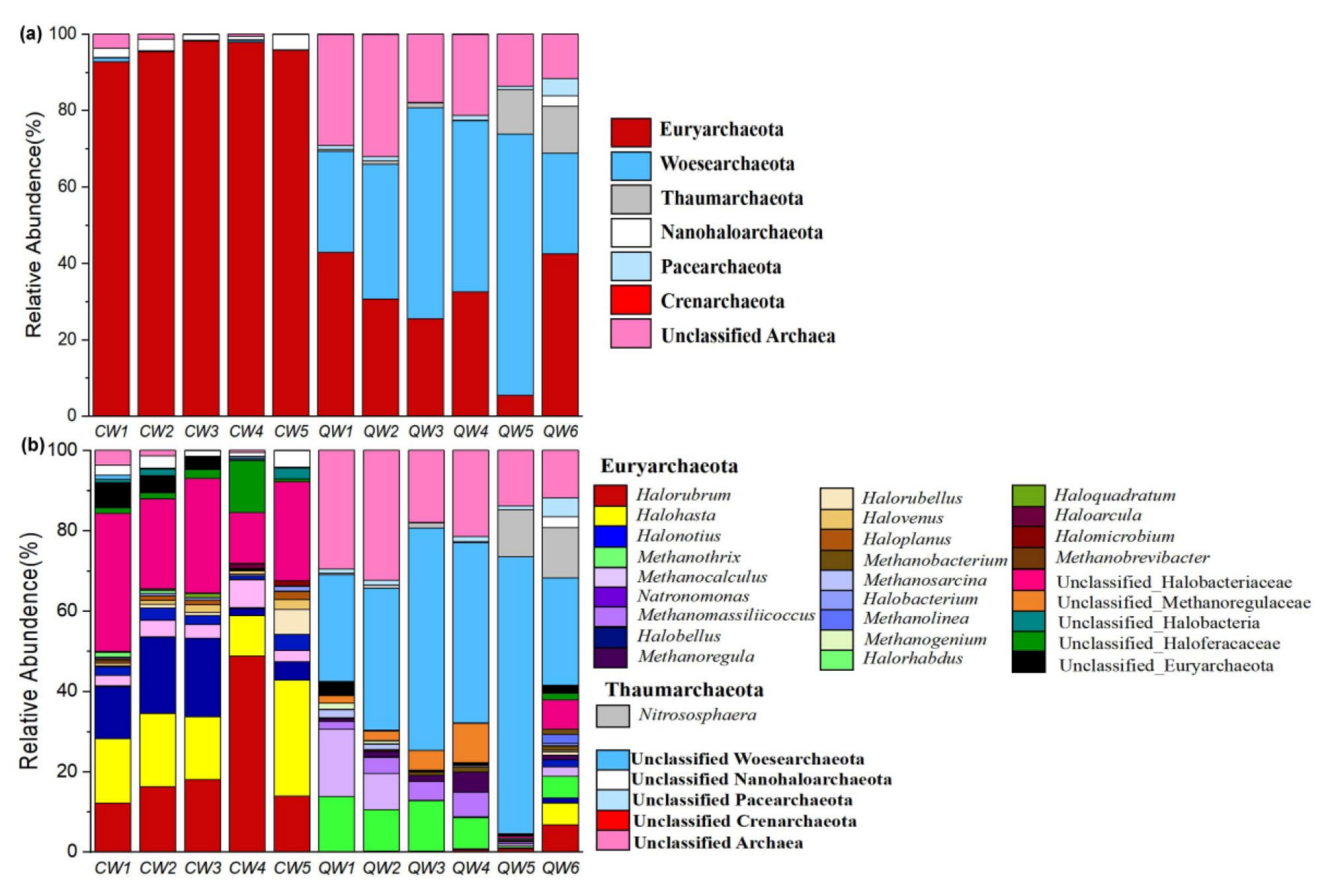
Relative abundance of archaea composition at phyla (**a**) and genus level (**b**) in various samples collected from the two lakes

At the phyla level, the relative abundance of archaeal sequences in Qinghai saline lake was dominated by Woesearchaeota (43.94%), followed by Euryarchaeota (29.16%) and Thaumarchaeota (4.8%) (Fig. 1a). By contrast, nearly all the sequences (95.74%) in Chaka hypersaline lake were annotated on Euryarchaeota. Except for Euryarchaeota, a few archaeal sequences in lake Chaka were also annotated on Nanohaloarchaeota (2.51%), Woesearchaeota (0.4%), and Thaumarchaeota (0.06%) (Fig. 1a). Notable, although phylum Euryarchaeota was dominant in both two lakes, at the genus level, the Euryarchaeota sequences in lake Qinghai and Chaka were mainly annotated on *Methanothrix*/ *Methanocalculus*/ *Methanomassiliicoccus* and *Halorubrum*/ *Halohasta*/ *Halonotius*/ *Natronomonas*, respectively (Fig. 1b). Thus, these evidences revealed archaea with markedly different taxonomic compositions in Qinghai saline lake and Chaka hypersaline lake.

**Fig. 2:**
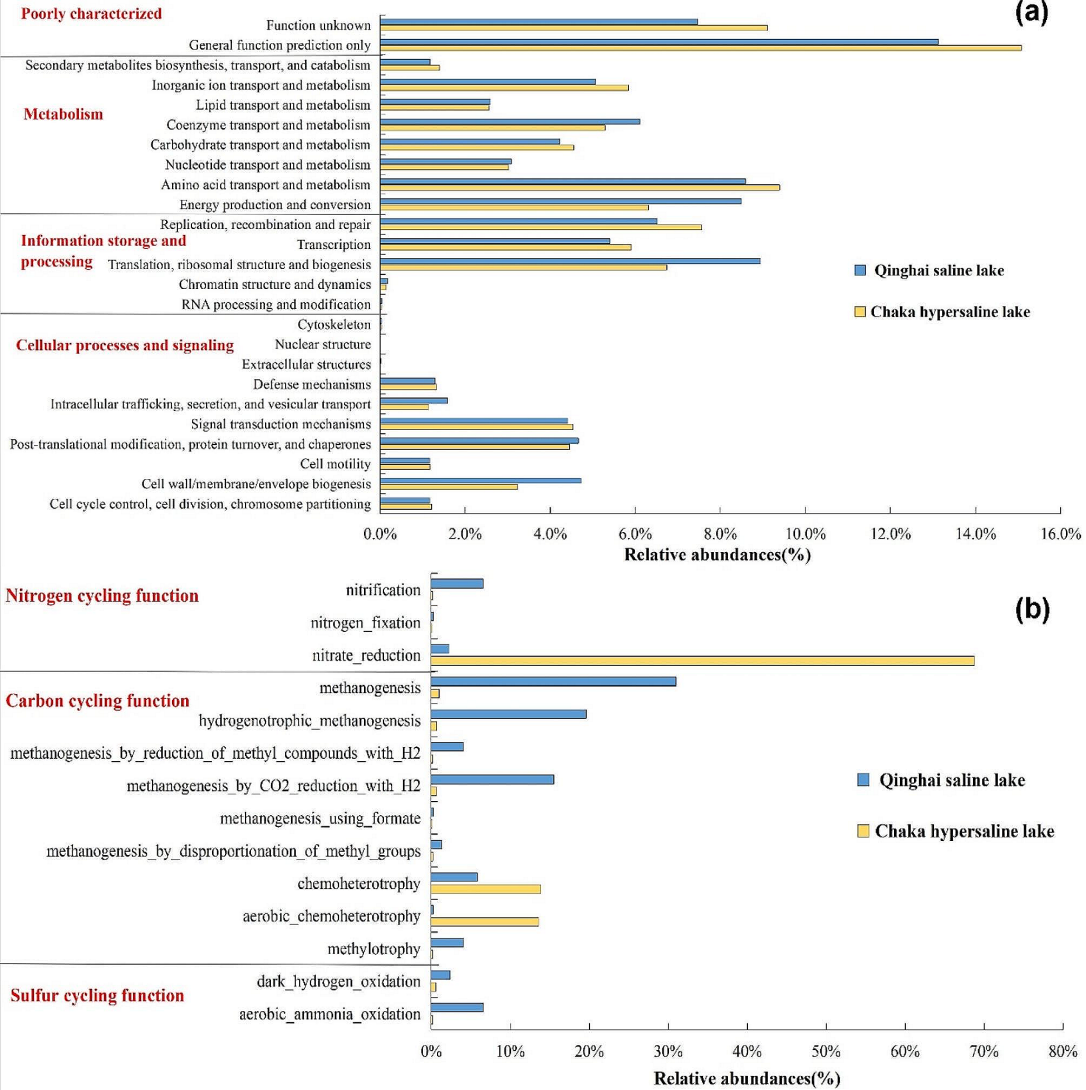
Functional annotation results of archaea in Qinghai saline lake and Chaka hypersaline lake. (**a**) metabolic pathways annotation (**b**) ecological function annotation

Using the PICRUSt software, we predicted the metabolic functions of archaea based on the COG database. In the Chaka hypersaline lake, archaeal metabolism was advantaged by ‘replication, recombination and repair’ and ‘inorganic ion transport metabolism’, which were some functions to tackle the extremely high-salinity environment. Comparatively, archaea in the Qinghai saline lake highly in ‘energy production and conversion’ and ‘translation, ribosomal structure and biogenesis’ (Fig. 2a). Furthermore, we utilized FAPROTAX to predict the ecological functions of the archaea communities in different habitats. Archaea in Qinghai saline lake showed a higher abundance of ‘methanogenesis’, ‘hydrogenotrophic methanogenesis’, and ‘methanogenesis by CO_2_ reduction with H_2_’ functions. In contrast, the archaea predominantly displayed ‘nitrate reduction’, ‘chemoheterotrophy’, and ‘aerobic chemoheterotrophy’ functionality in Chaka hypersaline lake (Fig. 2b).

### Impact of the environmental factors on archaeal communities

**Fig. 3:**
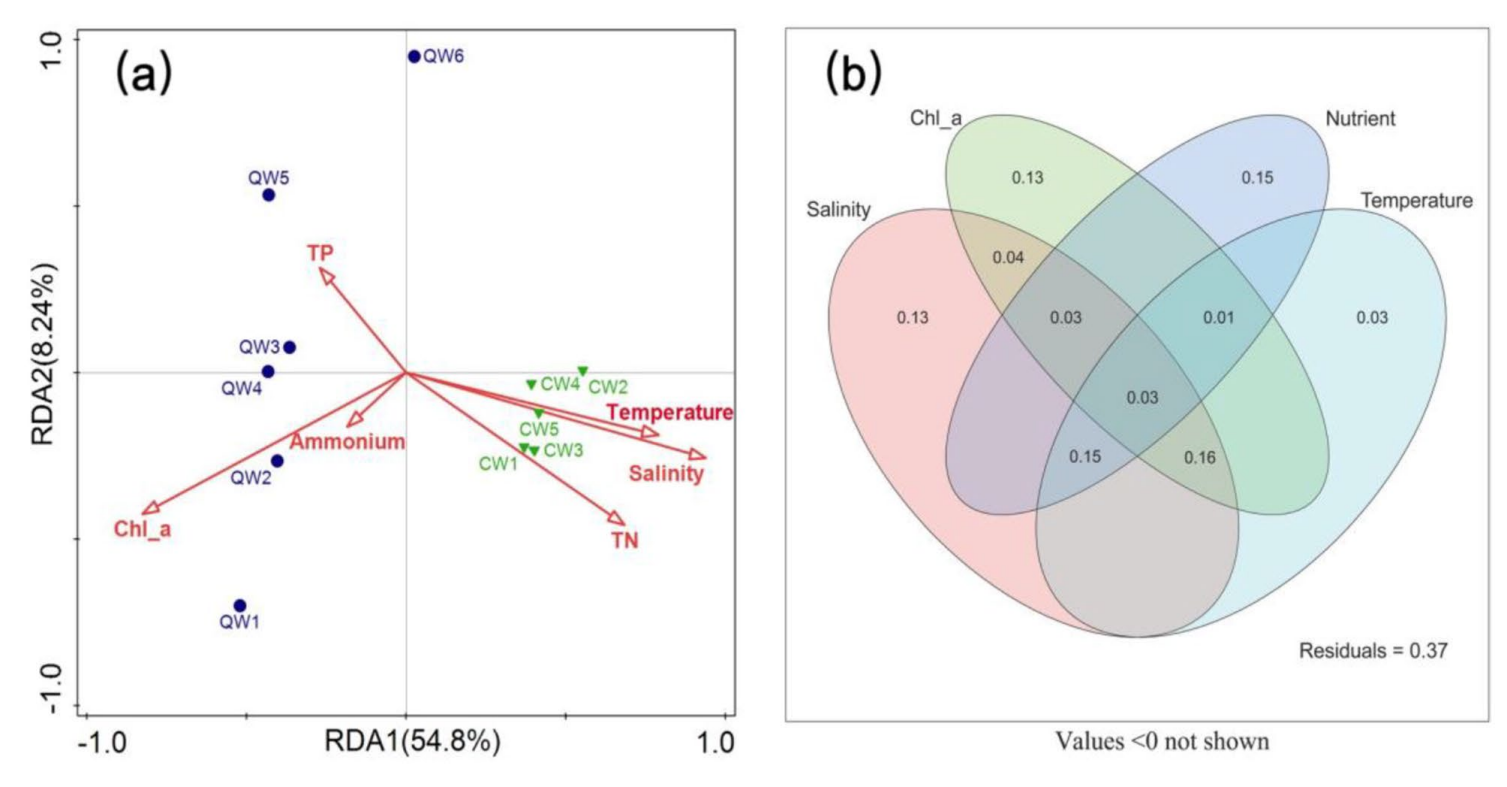
Archaeal communities’ variations across different habitats. (**a**) RDA analysis of archaea communities (**b**) VPA analysis of archaea communities

Following the exclusion with high inflation factors (> 10) (such as pH, NO_3_^−^ and DO), redundancy analysis (RDA) was used to understand the difference in the archaeal community structure of the two habitats and identify the significant environmental factors regulating their community structure. The RDA plot clearly demonstrated that the archaeal communities in the saline lake and hypersaline lake were substantially distinct (Fig. 3a). Salinity, Chlorophyll a (Chl_a), total nitrogen (TN), and temperature were identified as the most critical elements influencing the development of archaeal communities. Further variance partitioning analyses (VPA) confirmed that salinity largely impacted the LP community structure variation (Fig. 3b). But the contributions of Chl_a, temperature, and nutrient (TP + TN + PO_4_^3−^) to the archaeal communities’ variation were also unneglectable. In fact, archaeal variation explained by salinity combined temperature (representing 34% of the variation), Chl_a (representing 26% of the variation), or nutrient (representing 21% of the variation) was largely higher than the variation single explained by the salinity (represent 13% of the variation).

**Fig. 4:**
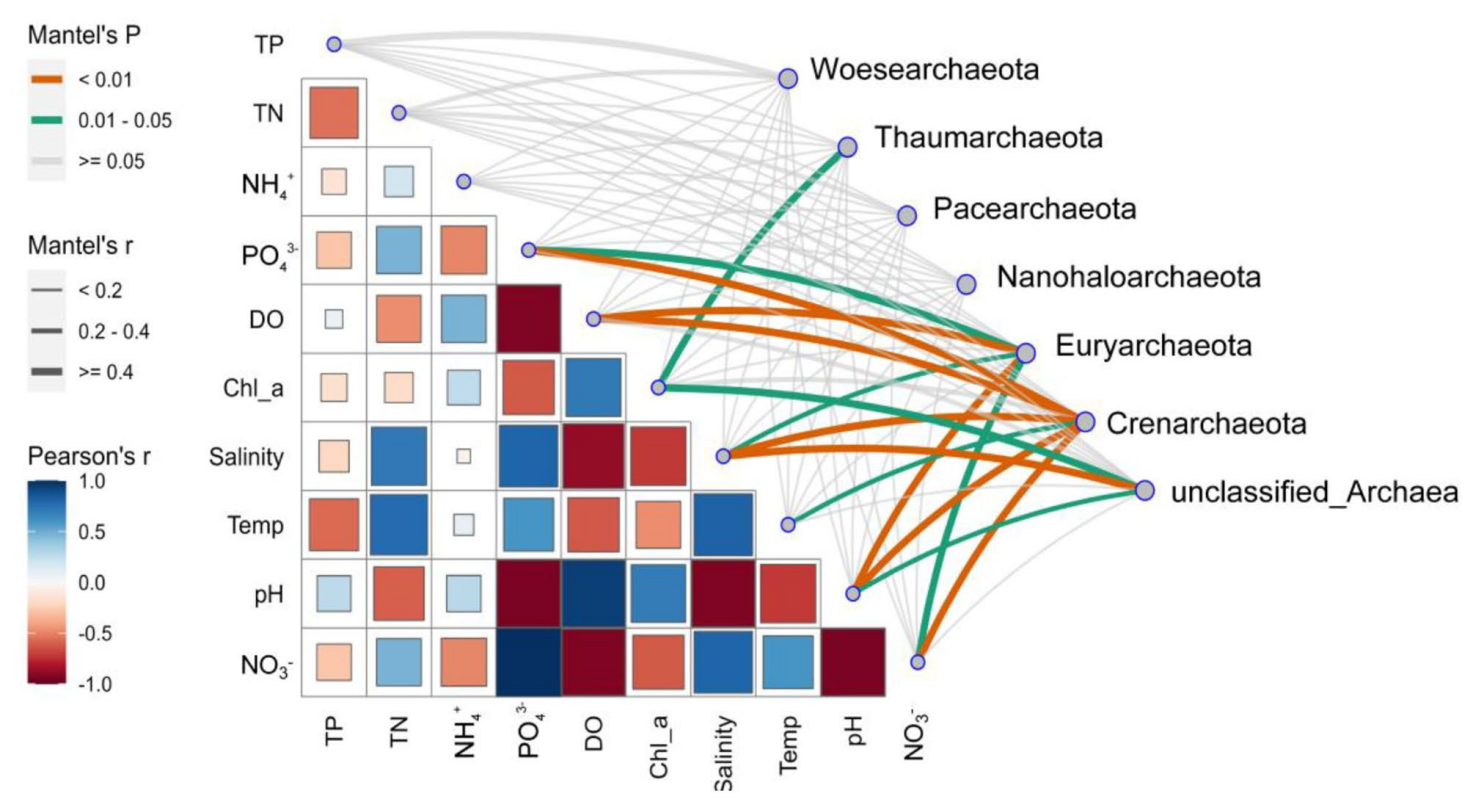
Mantel test for the effects of environmental variables on different phyla

The Mantel Test was used to examine the relationships between environmental conditions and the archaeal phyla. Consistent with prior findings, although salinity was found to significantly affect a lot of phyla, such as Euryarchaeota and Crenarchaeota, but it was not the only important factor affecting these phyla. Especially for the Euryarchaeota, which was one of the most important phyla to contribute to archaeal communities in both Chaka hypersaline and Qinghai saline lakes, DO, pH, and PO_4_^−^ were also found to have a large effect on them (Fig. 4).

### Archaeal interactions in different habitats and their response to environmental factors

**Fig. 5:**
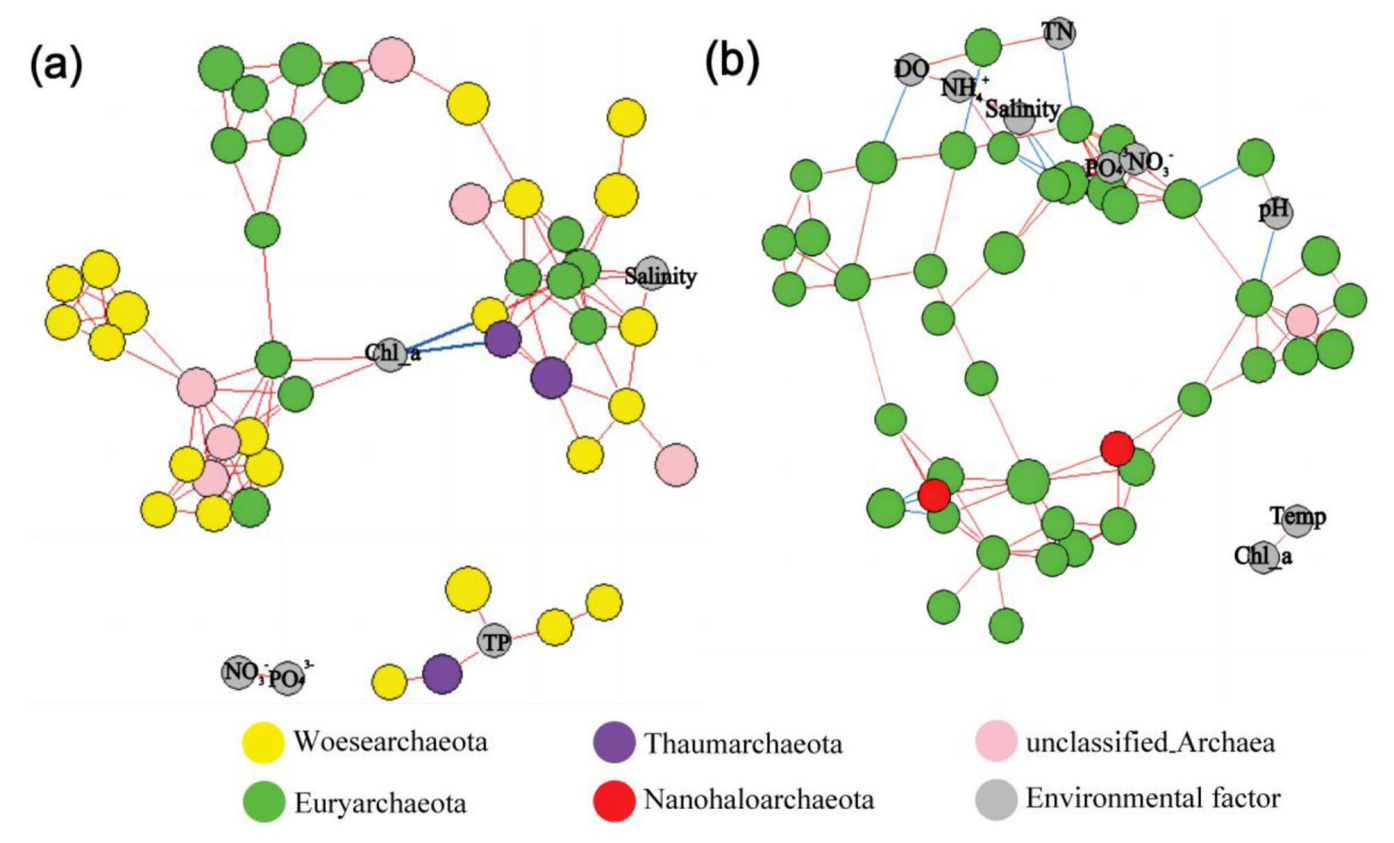
Dominant archaeal co-occurrence networks in Qinghai saline (**a**) and Chaka hypersaline (**b**) lakes. Positive and negative correlations were colored red and blue, respectively

Co-occurrence network analysis of the dominant archaeal OTUs (Top 50) was used to infer their interactions in different habitats. In Qinghai saline lake, the total nodes and correlation number of the network were 51 and 118, respectively (Fig. 5a). Surprisingly, the correlation between archaeal dominant OTUs in Qinghai saline lake was entirely positive, indicating the wide co-presence of the dominant archaea species. The number of the total nodes and correlation number of Chaka hypersaline lake were 53 and 120, respectively, which was not statistically different from those of Qinghai saline lake (Fig. 5b). However, some negative correlations between different dominant archaeal OTUs were found in Chaka hypersaline lake (∼ 8% of the total dominant archaeal OTUs’ correlations), representing the mutual exclusion existing in dominant archaeal OTUs under the extremely high salt environment.

Among the correlation relationships identified between dominant archaeal OTUs and environmental factors, there were also significant differences between the two habitats (Fig. 5). In the Qinghai saline lake, dominant archaeal OTUs were mainly correlated with salinity, total phosphorus (TP), and Chlorophyll a, but in the Chaka hypersaline lake, dominant archaeal OTUs were associated with DO, TN, pH, and PO_4_^3−^. Notable, although salinity was observed with correlation with dominant archaeal OTUs in both two habitats, their correlations’ positive/negative relationship was different. Specifically, the relationships between salinity and dominant archaeal OTUs were all positive in the network of Qinghai saline lake, but all negative in the network of Chaka hyper-saline lake. Moreover, in each habitat, the importance of some nutrient factors was higher than that of salinity. Especially, the number of correlations between dominant archaeal OTUs and phosphorus (TP/PO_4_^3−^) was equal to and exceeded that of salinity in the Qinghai and Chaka lakes, respectively.

## Discussion

In this study, the results of high-throughput sequencing revealed that the predominant phylum within the archaeal communities in Chaka hypersaline lake was Euryarchaeota (Fig. 1a). This observation was consistent with those of previous studies on the dominant archaeal phylum in the Great Salt Lake [28] and the Dead Sea [29]. Genus-level annotation further revealed Euryarchaeota in Chaka hypersaline lake was dominated by *Halorubrum, Halohasta, Halonotius and Natronomonas*, which was consistent with the archaeal community composition in high-altitude Andean lakes [30]. Based on culturable and genomic analysis, previous studies uncovered many *Natronomonas* and *Halorubrum* strains (e.g. *Natronomonas pharaonis* DSM 2160, and *Halorubrum lacusprofundi* ATCC 49,239) with the ability to reduce NO_3_^−^ and NO_2_^−^ assimilatorily [31, 32]. Interestingly, nitrate-reduction function was also found abundant in the hypersaline lake (Fig. 2b). Thus, these lines of evidence suggested archaea played important role in the nitrogen cycling of Chaka hypersaline lake. In the Qinghai saline lake, a previous culturable-dependent study supposed Euryarchaeota and Woesearchaeota were the first and second dominant phyla [33], respectively, which was different from the results of this study. Clearly, the most likely explanation for that variance was the difference in study methods. As a relatively new member of the superphylum DPANN (Diapherotrites, Parvarchaeota, Aenigmarchaeota, Nanoarchaeota, and Nanohaloarchaea), Woesearchaeota was surprisingly diverse and abundant in a wide range of extreme environments, such as deep oil reservoir, oligo-trophic lakes and indicating a high diversity of their roles in global biogeochemical cycles [34, 35]. Recently, multivariate regression analysis further revealed that Woesearchaeota might function in consortium with methanogens in the cycling of carbon [36]. Interestingly, our results also detected the wide co-presence of Woesearchaeota and methanogenesis-functional Euryarchaeota (Fig. 5a), which supported the previous observation. Furthermore, in the Qinghai saline lake, methanogenesis-related functions and taxa were distinctive characteristics of the archaeal annotation. Especially, the dominant genera in Qinghai saline lake were *Methanothrix* (average relative abundance > 8.30%) (Fig. 1b), which was widely distributed in both natural and artificial anoxic environments and played a major role in global methane production [37, 38]. Previous research has shown that some methanogenic archaea are halotolerant and can function with a salinity up to 20 g/L [39]. Thus, the appropriate salinity level (in an average of 16.5) and other environmental factors of Qinghai saline lake may suitable for the growth of these methanogenic archaea and allow them to gain some advantage in the communities. Considering the importance of methane in the greenhouse effect [40], the contribution of Qinghai saline lake to the greenhouse effect is worth further evaluating.

In this study, both network, RDA and Mantel-test results suggested the single influence of salinity on the structuring of archaeal communities was limited, which distinguished from prior study results that focused on the variation of bacterioplankton communities in different saline lakes [12]. This could be owing to the fact that many archaea have the ability to ‘live with salt’ [9]. Instead, the composite effect of salinity with diverse environmental parameters (e.g., temperature, chlorophyll a, total nitrogen, and total phosphorus) dominated the explanation of the variations in archaeal community structure. In oceanic habitats, several previous studies have suggested the structure of archaeal communities was significantly influenced by temperature, nitrogen, and chlorophyll-a concentrations [41, 42]. While in some freshwater lakes, a growing body of evidence suggested that phosphate or total phosphorus can essentially alter the structure of archaeal communities [43, 44]. The influence of environmental factors on archaea in saline lakes seems to be a combination of the two habitats mentioned above, but the specific regulatory effects of these factors and their mechanisms still need further analysis, especially based on culture-dependent studies and in situ investigations.

Although a lot of environmental factors have been considered, nearly 40% of the constrained variance of archaeal communities in different habitats was still unexplained, highlighting the limitations of employing environmental factors to explain community shifts. Currently, it has been reported that community structure predictions considering biotic information are more accurate than those based on only environmental factors [15, 45]. In this study, more than 80% of the dominant OTUs in each habitat correlated with others and the average correlation number of each note was greater than 4, indicating the strong internal relationships between archaea in each habitat. However, quantifying the contribution of archaeal interactions to their community shaping still remains a big challenge. Moreover, the proportion of positive/negative correlations of dominant archaeal OTUs was significantly different between the two habitats. In particular, negatively correlated relationships of dominant OTUs can only be detected in Chaka hypersaline lake. In Tibetan Plateau lakes, previous studies also uncovered the proportion of bacterial negative correlations increased from 21% in saline lakes to 43% in hypersaline lakes and postulated that high nutrient fluxes in well-connected habitats support the expression of competitive advantage and lead to the exclusion of inferior species [12]. According to our findings, the above theory may also apply to archaea communities.

## Conclusions

Using high-throughput sequencing, this study revealed the significantly different communities and potential ecological functions of archaea between Qinghai saline lake and Chaka hypersaline lake. Based on RDA, VPA, Mantel-test, and network analysis, we further suggested the differences of archaeal communities between saline and hypersaline lakes were driven by the combination of multiple environmental and non-environmental factors (archaeal interactions). Overall, this research improved our understanding of the structure and ecological role of archaea in saline lakes and provided an update on the mechanisms that shape their communities.

### Electronic supplementary material

Below is the link to the electronic supplementary material.

Supplementary Material 1

## Data Availability

Sequence data that support the findings of this study was deposited in the NCBI Sequence Read Archive (BioProject number PRJNA983497).
