# Supplementary material for: Differences in archaeal diversity and potential ecological functions between saline and hypersaline lakes on Qinghai-Tibet Plateau were driven by multiple environmental and non-environmental factors beyond the salinity
Source: BMC Microbiol. 2024 May 4;24:153. doi: 10.1186/s12866-024-03307-3 (PMC11069230; doi:10.1186/s12866-024-03307-3)

Table S1. Sampling stations and environmental parameters

| Number | Latitude | Longitude | TP（μg/L) | TN (mg/L) | NH_4_^+^  (mg/L) | NO_3_^-^  (mg/L) | PO_4_^3-^  (μg/L) | Salinity  (ppt) | pH | DO  (mg/L) | Chl_a  (μg/L) | Temperature  (℃) |
| --- | --- | --- | --- | --- | --- | --- | --- | --- | --- | --- | --- | --- |
| CW1 | 99°08′ | 36°74′ | 36.53 | 12.11 | 0.55 | 3.54 | 0.76 | 160.10 | 7.61 | 5.06 | 0 | 22.3 |
| CW2 | 99°08′ | 36°74′ | 2.32 | 14.29 | 0.35 | 3.42 | 0.02 | 160.00 | 7.64 | 5.03 | 1.674 | 28.4 |
| CW3 | 99°08′ | 36°74′ | 24.39 | 11.07 | 0.22 | 3.92 | 0.61 | 153.06 | 7.65 | 4.78 | 0 | 23.0 |
| CW4 | 99°08′ | 36°75′ | 31.01 | 14.48 | 1.24 | 1.02 | 0.33 | 177.76 | 7.91 | 5.79 | 0.534 | 26.0 |
| CW5 | 99°07′ | 36°75′ | 28.81 | 12.43 | 0.35 | 2.81 | 11.44 | 168.97 | 7.74 | 4.84 | 0.384 | 26.0 |
| Mean±sd |  |  | 24.61±10.8 | 12.88±1.31 | 0.542±0.37 | 2.94±1.03 | 2.63±4.41 | 163.98±8.54 | 7.71±0.11 | 5.10±0.36 | 0.518±0.615 | 25.1±2.2 |
| QW1 | 99°97′ | 36°66′ | 7.84 | 11.90 | 0.96 | 0.78 | 6.58 | 9.91 | 8.27 | 6.54 | 15.552 | 21.3 |
| QW2 | 100°01′ | 36°65′ | 29.91 | 9.25 | 0.53 | 0.69 | 6.58 | 9.58 | 8.16 | 6.29 | 9.721 | 18.3 |
| QW3 | 100°67′ | 36°54′ | 54.19 | 10.01 | 0.27 | 0.69 | 6.58 | 9.64 | 8.29 | 6.01 | 8.297 | 18.5 |
| QW4 | 100°64′ | 36°53′ | 23.29 | 9.76 | 0.68 | 0.69 | 6.58 | 10.21 | 8.22 | 6.45 | 10.253 | 22.6 |
| QW5 | 99°74′ | 36°82′ | 48.67 | 4.72 | 0.61 | 0.74 | 7.55 | 10.36 | 8.23 | 6.10 | 2.511 | 19.2 |
| QW6 | 99°97′ | 36°84′ | 23.29 | 9.70 | 0.53 | 0.78 | 6.58 | 10.42 | 8.24 | 6.49 | 1.674 | 20.4 |
| Mean±sd |  |  | 31.20±15.84 | 9.22±2.02 | 0.597±0.19 | 0.73±0.04 | 6.74±0.34 | 10.02±0.31 | 8.24±0.04 | 6.31±0.19 | 8.001±4.397 | 20.1±1.4 |

Based on the Wilcox.test, Salinity (*p*=0.004＜0.05), NO_3_^-^ (*p*=0.007＜0.05), and Temperature (Wilcox.test, *p*=0.014＜0.05) in the Chaka Lake was significantly higher than that of Qinghai Lake. While, pH (*p*=0.004＜0.05), DO (*p*=0.004＜0.05) and Chl_a (*p*=0.012＜0.05) in the Chaka Lake was significantly lower than that of Qinghai Lake. There were no significant differences in TP (Wilcox.test, *p*=0.927>0.05), NH_4_^+^ (Wilcox.test, *p*=0.521>0.05) and PO_4_^3-^ (Wilcox.test, *p*=0.104>0.05) between the two lakes.

Fig.S1 The principal coordinate (PCoA) analysis of archaeal communities in Qinghai saline lake and Chaka hypersaline lake.


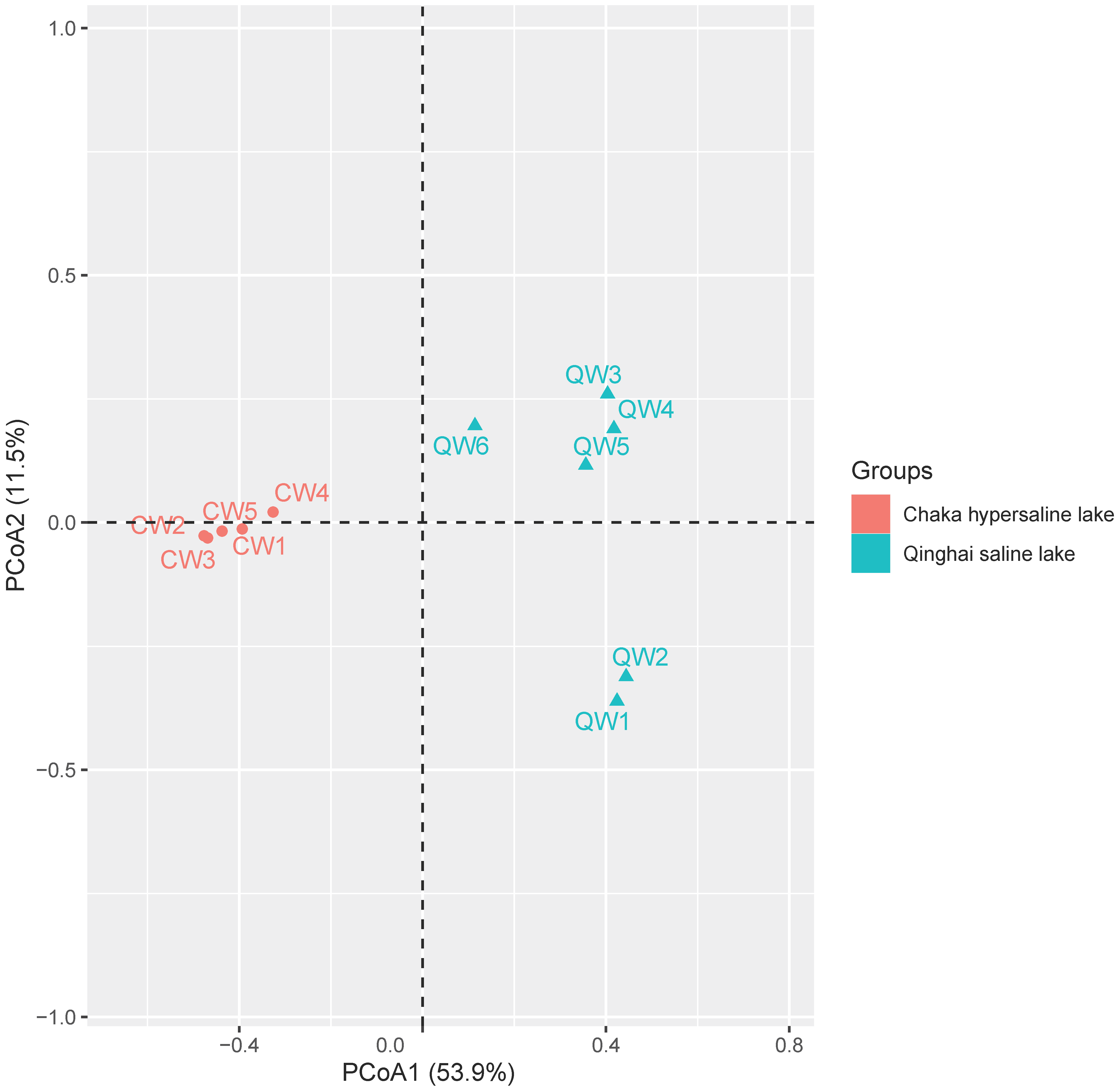

Supplement: Supplementary file 1 — Supplementary Material 1 [file 12866_2024_3307_MOESM1_ESM.docx]
